# Acute and chronic toxicity of a polyherbal preparation – Jueyin granules

**DOI:** 10.1186/s12906-018-2211-z

**Published:** 2018-05-08

**Authors:** Yu Chen, Dong-jie Guo, Hui Deng, Min-feng Wu, Ya-Nan Zhang, Su Li, Rong Xu, Jie Chen, Xing-xiu Jin, Bin Li, Qi Xu, Fu-lun Li

**Affiliations:** 1 Department of Dermatology, Yueyang Hospital of Integrated Traditional Chinese and Western Medicine, affiliated with Shanghai University of Traditional Chinese Medicine, 110 Ganhe Road, Shanghai, 200437 China; 2 Department of Dermatology, the Seventh People’s Hospital of Integrated Traditional Chinese and Western Medicine, affiliated with Shanghai University of Traditional Chinese Medicine, Shanghai, 200137 China; 3 The Sixth Hospital Affiliated with Shanghai Jiaotong University, Shanghai, 200233 China; 4 School of Public Health, Shanghai University of Traditional Chinese Medicine, Shanghai, 200433 China

**Keywords:** Jueyin granules, Acute toxicity, Chronic toxicity

## Abstract

**Background:**

The potential toxicity of Chinese herbal medicine has attracted more attention in recent years**.** Jueyin granules (JYG), a polyherbal formula, have been proven to be an effective agent for treating psoriasis in both animal models and clinical research. However, little is known about the possible acute and chronic toxicity of JYG. The objective of this study was to investigate the safety of JYG in ICR mice and Wistar rats.

**Methods:**

To examine the acute toxicity of JYG, ICR mice were randomly divided into an experimental group and a control group, each comprising 20 mice (10 male and 10 female). The experimental group was fed JYG solution at a dose of 21.5 g/kg, equivalent to 143 times the clinical human dosage, for 14 days, whereas control animals were fed distilled water. In the chronic toxicity test, Wistar rats were divided into four groups, each comprising 40 rats (20 male and 20 female). For 6 months, the experimental animals were given JYG at a dose of 7.5, 3.75 and 1.875 g/kg, whereas control animals were given distilled water. The animals’ body weight, food and water consumptions were monitored weekly. In addition, their biochemical and hematological parameters, histopathology, and body and organ weights were all measured at specific observation time points.

**Results:**

According to the results of the acute toxicity test, no mortality was found and no abnormal pathological changes in major organs were observed in mice treated with JYG. In the chronic toxicity test, JYG did not cause significant abnormalities in the physiological parameters or pathological changes in the major organs of the rats.

**Conclusion:**

The results indicated that JYG at the given doses did not induce any harmful effects in animals. Thus, it is reasonable to conclude that JYG is safe at the studied dosage levels and causes no acute or chronic toxicity in animal models.

**Electronic supplementary material:**

The online version of this article (10.1186/s12906-018-2211-z) contains supplementary material, which is available to authorized users.

## Background

Although traditional medicine provides front-line pharmacotherapy for millions of Chinese, its application is often viewed with skepticism by the Western medicine establishment [1]. There has been wide concern about the toxicity of herbal medicine, and several side effects (such as allergic reactions, hepatotoxicity, nephrotoxicity, and cardiac toxicity) of herbal medicines have been reported in recently years [2].

Psoriasis is a chronic inflammatory skin disease affecting more than 125 million people worldwide [3]. Currently, there is no cure for psoriasis. Patients with psoriasis in China often turn to alternative and complementary treatments, which are considered to be effective and safe [4]. Jueyin granules (JYG), an effective formula consisting of eight Chinese herbs (*Haliotis diversicolor, Flos Lonicerae Japonicae, Radix Rehmanniae* exsiccate*,* cortex moutan, *Herba Hedyotisdiffusae, Folium isatidis, Smilax china L. and Radix Curcumae*) were discovered in the 1950s by Han Xia (a well-known Chinese surgeon) and have been used to clinically treat psoriasis for over 50 years by Yueyang Hospital of Integrated Traditional Chinese and Western Medicine. Our previous study showed that JYG can reduce inflammation and proliferation of keratinocytes and prevent psoriasis in animal models [5]. Moreover, the major ingredients, including *Haliotis diversicolor, Flos Lonicerae Japonicae, Herba Hedyotis diffusae, Folium Isatidis, Smilax china L., Radix Curcumae,* have been demonstrated to have anti-inflammatory effects in vitro and in vivo models [6–12]. The ingredient *Cortex Moutan* has been reported to have an inhibitory effect on proliferation of HaCaT cells in vitro models [13]. However, the toxicity of JYG has not been well studied. The objective of this study was to evaluate the safety of JYG in animal models.

## Methods

### Testing materials

Jueyin granules(manufactured by Tianyin Pharmaceutical Co. Ltd., Jiangsu Province, China; Certified Number of 20,120,103) were prepared using a water–alcohol extraction method and its quality control was performed using high-performance liquid chromatography (HPLC) by detecting chlorogenic acid and paeonol as shown in a previous publication [5]. The suspension of the drug was prepared by purified water. Its composition is shown in Table 1.

**Table 1:** Ingredients of JYG used with English translations

Medicine	English translation	species/ family	dose
*Haliotis diversicolor*	Concha Haliotidis	Haliotis diversicolor Reeve	15 g
*Flos Lonicerae japonicae*	Honeysuckle flower	Lonicera japonica Thunb	12 g
*Radix Rehmanniae exsiccata*	Dried Rehmannia root	Rehmannia glutinosa(Gaertn.)Libosch	15 g
*Cortex Moutan*	Tree peony bark	Paeonia suffruticosa Andr	12 g
*Herba Hedyotisdiffusae*	Oldenlandia	Hedyotis diffusa Willd	15 g
*Folium isatidis*	Dyer’s woad leaf	Isatis indigotica Fort	15 g
*Smilax china L.*	Chinaroot greenbrier rhizome	Smilaz china L	15 g
*Radix Curcumae*	Turmeric root tuber	Curcuma longa L	9 g

### Animals

ICR mice weighing 17.2–19.8 g, purchased from Shanghai Super B & K Laboratory Animal Corp., Ltd., were used for the acute toxicity test. Six-to-seven-week-old SPF grade Wistar rats, purchased from Beijing WeiTongLiHua Experimental Animal Technology Co., Ltd., were used for the chronic toxicity test (Animal certificate no. 11400700011308). All animals were housed in groups of five rats per cage under a schedule of 12 h light/12 h dark and in a controlled temperature of 21 °C–24 °C. Animals had free access to standard laboratory animal feed and water. The experimental protocols were approved by the institutional Animal Ethics Committee of Shanghai University of Traditional Chinese Medicine (No. 14480 and 14,486).

### Testing methods

#### Acute toxicity test

Forty ICR mice were randomly divided into two groups, each comprising 10 males and 10 females. For 18 h before the start of the experiment, the mice had access to water but no food. The mice were fed JYG oral solution, 40 ml/kg, by gavage twice a day for 14 days. The animals’ skin, mucous membrane, changes in fur color, eyes, circulation, central nervous system, respiration, and conscious behavior were observed daily. Body weights were also measured once a week. Mice were euthanasized with CO2 inhaltion on the 14th day.

#### Chronic toxicity test

A total of 160 Wistar rats were randomly divided into four groups, each group comprising 40 animals of 20 males and 20 females. All rats in the experimental groups were fed JYG oral solution once a day at graded doses of 7.5 (JYG-H), 3.75 (JYG-M), and 1.875 (JYG-L) g/kg for 3 months and 6 months, respectively, followed by a recovery period of 4 weeks. Those in the control group were administered distilled water at 20 ml/kg/d. The rats were observed daily for abnormal behavior and other adverse signs of toxicity. Consumption of food and water as well as body weight were recorded weekly. All animals were euthanasized with CO2 inhaltion at the end of testing, and blood samples were obtained for the biochemical assays. The liver, kidney, lung, heart, spleen, brain, ovaries, testes and adrenal gland were all collected, weighed, and homogenized. A portion of each organ was removed for histological studies.

### Biochemical assay

#### Hematological assessments

Hematological parameters, such as total white blood cell count, red blood cell count, packed cell volume, hemoglobin, and platelets (PLT), were determined using a fully automated hematology analyzer (Simens, Bayer ADVIA120, Germany).

#### Liver, renal function and serum electrolytes tests

A Hitachi 7020 Automatic Biochemical Analyzer was used to detect aspartate aminotransferase (AST), alanine aminotransferase (ALT), alkaline phosphatase, blood urea nitrogen, total protein, albumin, blood glucose, total bilirubin, creatinine, total cholesterol, triglycerides, creatine kinase, sodium ion concentration, potassium ion concentration, and chloride ion concentration.

#### Histopathology

The brain, liver, spleen, adrenal gland, epididymis, uterus, heart, kidney, testis, ovary, lung, and thymus were all weighed to calculate organ coefficients. The thyroid, stomach, pancreas, testis, prostate, aorta, bladder and bone marrow were preserved in 10% Faure Marin solution, fixed for 36–48 h, and subjected to conventional histological processes for histopathological examination. The tissue sections were examined under a microscope with a 40× objective to check cell morphology and quantity.

### Statistical analysis

The experimental data were analyzed using SPSS 21 statistical software. All data were expressed as the mean ± the standard error of the mean (SEM).Significant differences among the groups were determined by a one-way analysis of variance and post hoc testing was performed for inter-group comparisons for least significant differences (LSDs) using a statistical analysis program for social science (SPSS)*.*

## Results

### Acute toxicity

The results of acute oral toxicity testing of JYG administered at the dose of 21.5 g/kg are shown in Table 2. No animal mortality occurred at the doses given, and no signs of abnormality were observed throughout the experiment. The weight of the control group and the experimental group were both increased, and the average weight of those groups had no significant difference. In addition, no histopathological changes were observed in either the control or the JYG-treated groups (Additional file 1: Figure S1-S2).

**Table 2:** Body weight in the control and JYG-treated group in the acute toxicity test

Group	Dose (g/kg)	n	Body weight (X ± S)		
			Day0	Day7	Day14
Control	/	20	18.7 ± 0.8	27.9 ± 1.9	31.0 ± 2.8
JYG	21.5	20	18.5 ± 0.7	26.4 ± 2.6	30.5 ± 3.6

*JYG* Jueyin granules. Values are expressed as mean ± SEM. JYG-treated groups showed non-significant changes as compared with control mices (*P* > 0.05)

### Chronic toxicity

#### General conditions

Following the oral administration of JYG, rats from the low, medium, and high dose groups as well as those in the control group, were all in good condition. There were no significant abnormalities in fur color, behavior, eating, drinking, or breathing, nor were there any abnormalities in secretions from the eyes, mouths, noses, or other cavities. Eight rats died because of operational errors (Additional file 1: Figure S3-S4).

#### Effect of JYG on body weight, food consumption, organ weight and relative organ weight

**Fig. 1:**
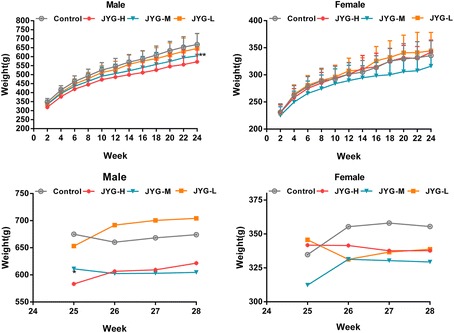
Body weight in the control and JYG-treated groups in the chronic toxicity test. Note: JYG-H, JYG-M, JYG-L: Jueyin granules high-dose, medium dose, low-dose group, respectively. The values are expressed as mean ± SEM (*n* = 10 rats for 3 months; *n* = 17–19 rats for 6 months; n = 10 rats for 1 month recovery). * *P* < 0.05; **P < 0.01 statistically significant compared to control group

**Fig. 2:**
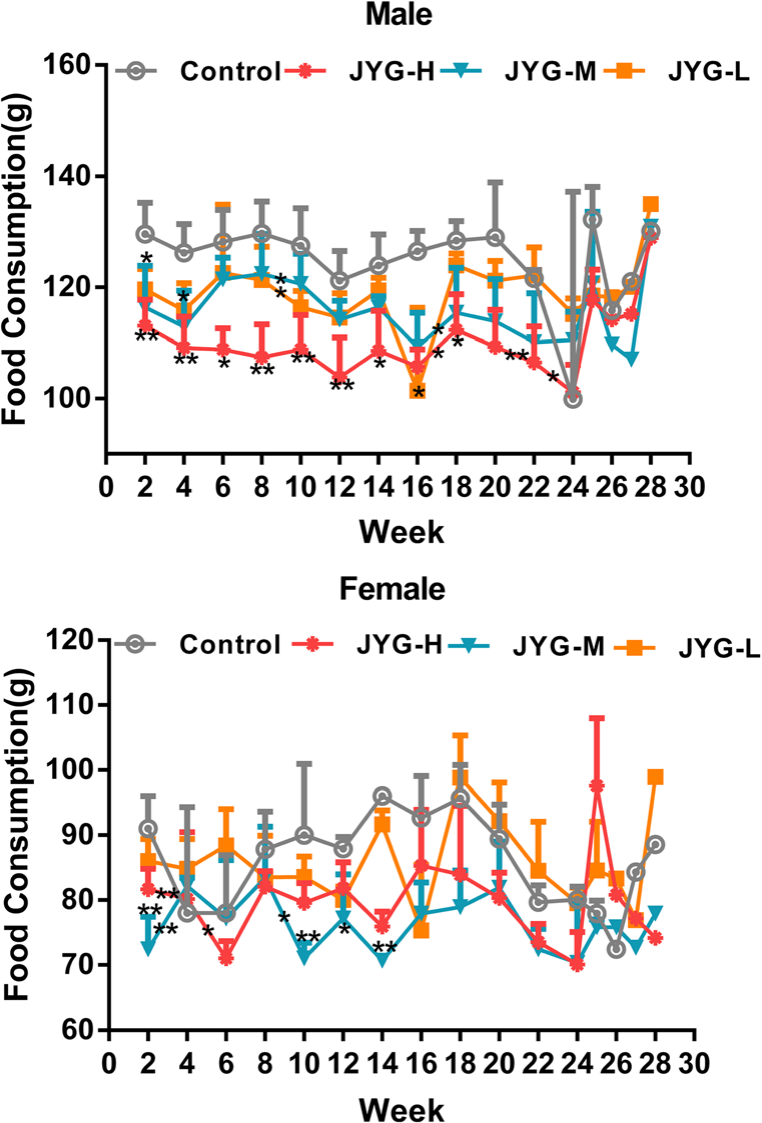
Food consumption in the control and JYG-treated groups in the chronic toxicity test. Note: JYG-H, JYG-M,JYG-L: Jueyin granules high-dose, medium dose, low-dose group, respectively. The values are expressed as mean ± SEM (*n* = 10 rats for 3 months; *n* = 17–19 rats for 6 months; n = 10 rats for 1 month recovery). * *P* < 0.05; ** *P* < 0.01 statistically significant compared to control group

**Fig. 3:**
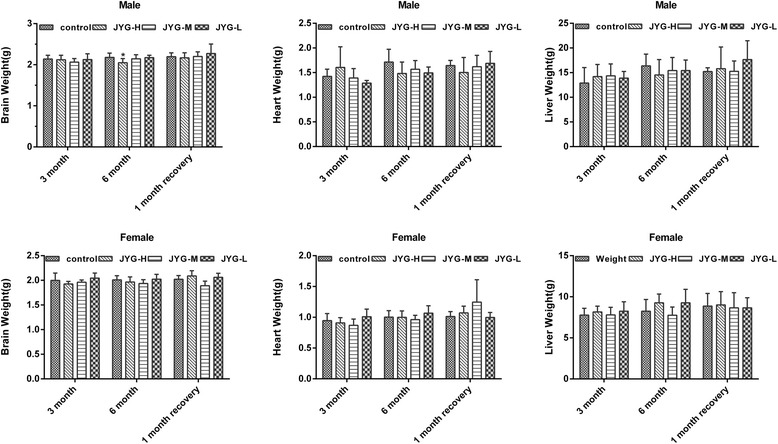
Organs weight of brain, heart and liver in the control and JYG-treated groups in the chronic toxicity test. Note: JYG-H, JYG-M,JYG-L: Jueyin granules high-dose, medium dose, low-dose group, respectively. The values are expressed as mean ± SEM (*n* = 10 rats for 3 months; *n* = 17–19 rats for 6 months; n = 10 rats for 1 month recovery). * *P* < 0.05 statistically significant compared to control group

**Fig. 4:**
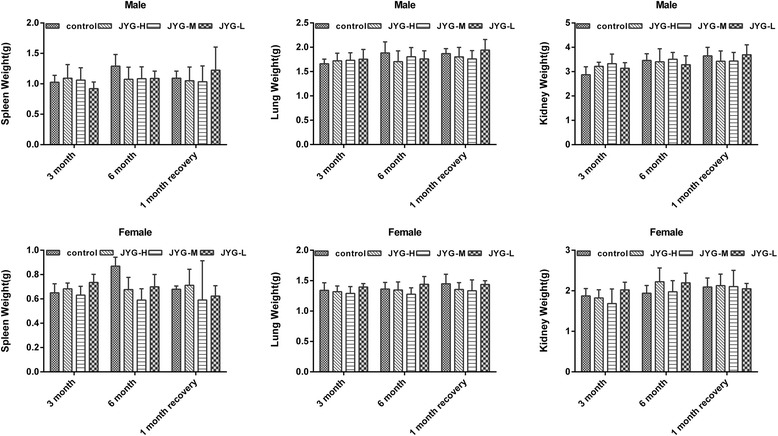
Organs weight of spleen, lung, and kidney in the control and JYG-treated groups in the chronic toxicity test. Note: JYG-H, JYG-M,JYG-L: Jueyin granules high-dose, medium dose, low-dose group, respectively. The values are expressed as mean ± SEM (n = 10 rats for 3 months; n = 17–19 rats for 6 months; n = 10 rats for 1 month recovery). JYG-treated groups showed non-significant changes as compared with control group (*P* > 0.05)

**Fig. 5:**
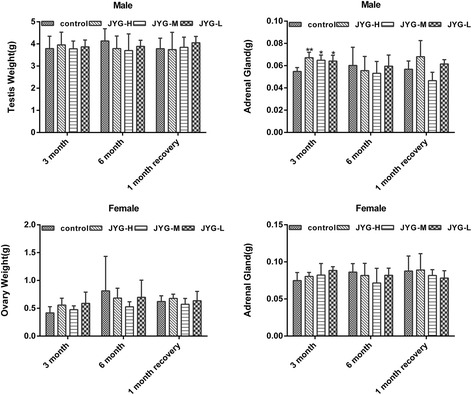
Organs weight of testis, ovary, and adrenal gland in the control and JYG-treated groups in the chronic toxicity test. Note: JYG-H, JYG-M,JYG-L: Jueyin granules high-dose, medium dose, low-dose group, respectively. The values are expressed as mean ± SEM (n = 10 rats for 3 months; n = 17–19 rats for 6 months; n = 10 rats for 1 month recovery). * *P* < 0.05; ** *P* < 0.01 statistically significant compared to control group

**Fig. 6:**
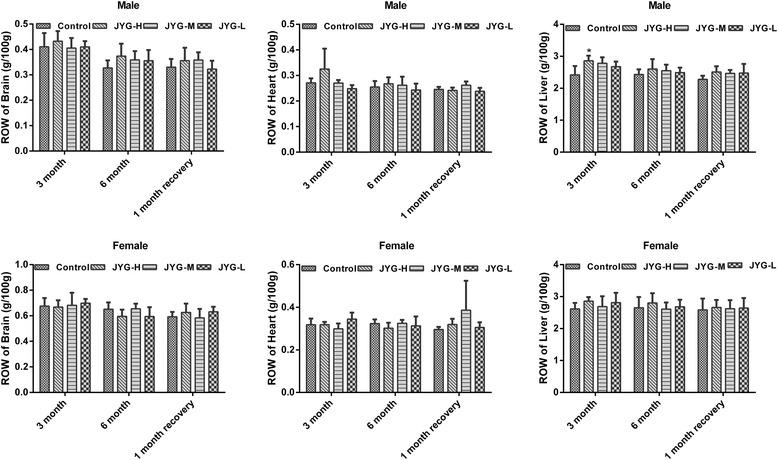
Relative organs weight of brain, heart and liver in the control and JYG-treated groups in the chronic toxicity test. Note: ROW: relative organs weight; JYG-H, JYG-M, JYG-L: Jueyin granules high-dose, medium dose, low-dose group, respectively. The values are expressed as mean ± SEM (n = 10 rats for 3 months; n = 17–19 rats for 6 months; n = 10 rats for 1 month recovery). * *P* < 0.05 statistically significant compared to control group

**Fig. 7:**
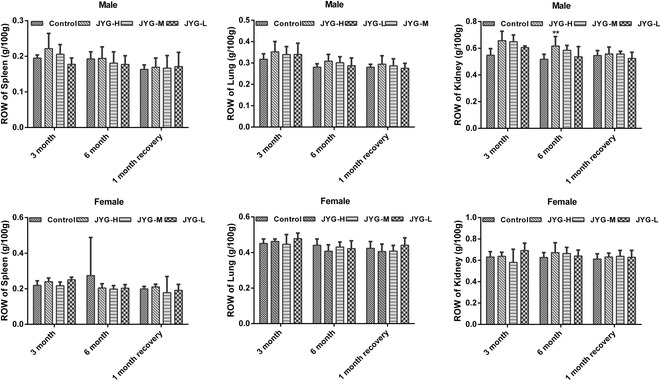
Relative organs of spleen, lung, and kidney in the control and JYG-treated groups in the chronic toxicity test. Note: ROW: relative organs weight; JYG-H, JYG-M, JYG-L: Jueyin granules high-dose, medium dose, low-dose group, respectively. The values are expressed as mean ± SEM (n = 10 rats for 3 months; n = 17–19 rats for 6 months; n = 10 rats for 1 month recovery). ** *P* < 0.01 statistically significant compared to control group

**Fig. 8:**
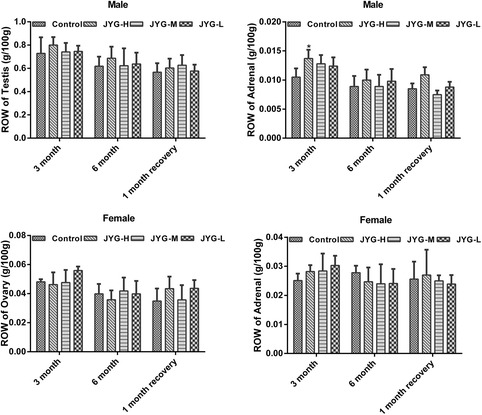
Relative organs of testis, ovary, and adrenal gland in the control and JYG-treated groups in the chronic toxicity test. Note: ROW: relative organs weight; JYG-H, JYG-M, JYG-L: Jueyin granules high-dose, medium dose, low-dose group, respectively. The values are expressed as mean ± SEM (n = 10 rats for 3 months; n = 17–19 rats for 6 months; n = 10 rats for 1 month recovery). * *P* < 0.05 statistically significant compared to control group

Compared with the control group, JYG-H male rats had lower weight from weeks 2 to 24, although slow appreciable growth was observed at weeks 1, 2, and 18. JYG-M females had lower weight at week 2 (see Fig. 1). In most weeks from week 1 to 23, JYG-H male rats took in less food (except during weeks 3, 15, 16, 18–20, and 22). JYG-M male rats consumed less food during weeks 1, 2, 3, 5, 9, and 17, and JYG-M female rats consumed less food during weeks 1, 2, 3, 9, 10, and 12–14. JYG-L male rats ate less food during weeks 9, 16, and 17 (see Fig. 2). No differences were found in average bodyweight and food intake throughout the six-month experimental period at the other time points. The brain weight of JYG-H male rats was lower and kidney relative weight was heavier at the end of 6 months compared with the control group. The liver relative weight of JYG-H male rats was heavier at the end of 3 months compared with the control group. The adrenal gland weight of male rats in JYG-treated groups was heavier than that of the control group. No significant differences were found in other organ weight values (see Figs. 3, 4, 5, 6, 7 and 8).

#### Effect of JYG on hematology parameters and biochemistry parameters

**Fig. 9:**
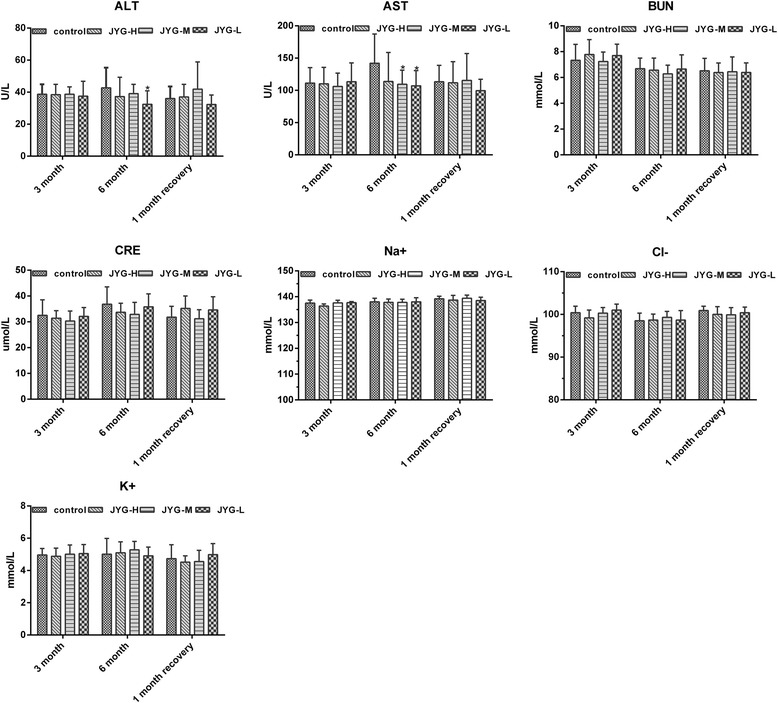
Biochemical parameters in the control and JYG-treated groups in the chronic toxicity test. Note: JYG-H, JYG-M, JYG-L: Jueyin granules high-dose, medium-dose, low-dose group, respectively; ALT: alanine aminotransferase; AST: aminotransferase; BUN: blood urea nitrogen; CRE: creatinine; Na^+^: Sodium ion; Cl^−^: Chloride ion; K^+^: Potassium ion. The values are expressed as mean ± SEM (n = 10 rats for 3 months; n = 17–19 rats for 6 months; n = 10 rats for 1 month recovery). * *P* < 0.05 statistically significant compared to control group

In female rats, JYG-H rats had significantly lower RBC and HGB values at the third months. In male rats, JYG-H and JYG-M rats had lower RBC values at the end of 6 months, whereas HGB values were significantly higher in JYG-H rats after 1 month of treatment in comparison to control group. By the sixth month, a significant decrease in Mono values was noted in low dose compared to the control group (see Tables 3 and 4). At the end of 6 months, JYG-L rats had significantly lower ALT values, whereas AST values was significantly lower in JYG-M and JYG-L rats compared to the control group (see Fig. 9). However, these values were still within the normal range. There was no significant difference in other hematological and biochemistry parameters between the JYG-treated and the control groups.

**Table 3:** Hematological parameters of female rats in chronic toxicity test

Treaments	Time	Control	JYG-H	JYG-M	JYG-L
			7.5 g/kg	3.75 g/kg	1.875 g/kg
WBC	3 month	3.99 ± 1.44	3.46 ± 0.58	3.81 ± 0.49	4.04 ± 2.26
(× 109/L)	6 month	2.41 ± 1.31	1.95 ± 0.42	2.00 ± 0.79	1.90 ± 0.32
	1 month recovery	4.58 ± 1.82	4.86 ± 1.96	4.42 ± 2.22	4.43 ± 0.88
RBC	3 month	7.99 ± 0.42	7.38 ± 0.28*	7.84 ± 0.29	7.91 ± 0.48
(× 1012/L)	6 month	7.55 ± 1.19	7.71 ± 0.25	7.84 ± 0.52	8.03 ± 0.42
	1 month recovery	8.60 ± 0.35	8.35 ± 0.79	8.57 ± 0.44	8.41 ± 0.28
HGB	3 month	15.26 ± 0.61	14.40 ± 0.44*	14.86 ± 0.54	15.24 ± 0.86
(g/L)	6 month	14.30 ± 2.11	14.66 ± 0.89	14.64 ± 0.82	15.00 ± 0.43
	1 month recovery	15.24 ± 0.85	15.24 ± 0.68	15.50 ± 0.88	14.86 ± 0.60
PLT	3 month	1264 ± 54	1254 ± 210	1196 ± 106	1207 ± 172
(× 109/L)	6 month	1209 ± 347	1099 ± 120	1128 ± 110	1109 ± 156
	1 month recovery	1195 ± 140	1210 ± 198	1179 ± 98	1137 ± 121
NEUT	3 month	0.91 ± 0.62	0.98 ± 0.15	0.91 ± 0.13	0.94 ± 0.54
(× 109/L)	6 month	0.84 ± 0.57	0.66 ± 0.24	0.58 ± 0.16	0.58 ± 0.14
	1 month recovery	1.45 ± 0.58	1.65 ± 0.59	1.13 ± 0.65	1.30 ± 0.30
LYMPH	3 month	2.67 ± 1.08	2.15 ± 0.52	2.57 ± 0.40	2.77 ± 1.55
(× 109/L)	6 month	1.14 ± 0.47	1.10 ± 0.25	1.17 ± 0.54	1.11 ± 0.22
	1 month recovery	2.72 ± 1.28	2.75 ± 1.55	2.76 ± 1.57	2.69 ± 0.78
MONO	3 month	0.19 ± 0.10	0.22 ± 0.08	0.18 ± 0.07	0.20 ± 0.13
(× 109/L)	6 month	0.14 ± 0.13	0.09 ± 0.04	0.13 ± 0.13	0.11 ± 0.05
	1 month recovery	0.18 ± 0.12	0.23 ± 0.15	0.19 ± 0.15	0.22 ± 0.14

*JYG-H, JYG-M,JYG-L* Jueyin granules high-dose, medium dose, low-dose group, respectively. *WBC* total white blood cell count, *RBC* red blood cell, *HGB* hemoglobin, *PLT* blood platelet, *Neut* neutrophil, *Lymph* lymphocyte, *Mono* mononucleosis. The values are expressed as mean ± SEM (*n* = 10 rats for 3 months; *n* = 17–19 rats for 6 months; n = 10 rats for 1 month recovery). * Significantly different from control group (*p* < 0.05)

**Table 4:** Hematological parameters of male rats in chronic toxicity test

Treaments	Time	Control	JYG-H	JYG-M	JYG-L
			7.5 g/kg	3.75 g/kg	1.875 g/kg
WBC	3 month	5.00 ± 1.01	8.27 ± 3.50	5.82 ± 1.28	6.81 ± 1.10
(× 109/L)	6 month	6.23 ± 2.20	6.06 ± 3.20	5.55 ± 1.23	5.52 ± 1.64
	1 month recovery	2.44 ± 1.30	2.36 ± 1.05	2.78 ± 0.85	2.33 ± 1.21
RBC	3 month	8.42 ± 0.67	8.06 ± 0.23	8.37 ± 0.16	8.58 ± 0.21
(× 1012/L)	6 month	8.80 ± 0.43	8.16 ± 0.51*	8.33 ± 0.47*	8.70 ± 0.39
	1 month recovery	7.05 ± 0.65	7.92 ± 0.76	7.57 ± 0.53	7.06 ± 0.79
HGB	3 month	14.56 ± 0.68	14.76 ± 0.36	15.02 ± 0.23	15.06 ± 0.42
(g/L)	6 month	15.14 ± 0.37	14.51 ± 0.78	14.72 ± 0.76	15.21 ± 0.49
	1 month recovery	13.26 ± 1.14	15.10 ± 1.01*	14.18 ± 1.09	13.14 ± 1.38
PLT	3 month	1214 ± 164	1108 ± 132	1080 ± 79	1186 ± 83
(× 109/L)	6 month	1134 ± 172	1182 ± 202	1102 ± 213	1210 ± 114
	1 month recovery	855 ± 314	897 ± 185	814 ± 238	759 ± 399
NEUT	3 month	1.53 ± 0.65	2.61 ± 1.69	1.51 ± 0.76	1.91 ± 0.70
(× 109/L)	6 month	1.61 ± 0.56	2.01 ± 1.13	1.71 ± 0.47	1.65 ± 0.81
	1 month recovery	0.74 ± 0.31	0.67 ± 0.20	0.93 ± 0.08	1.09 ± 0.86
LYMPH	3 month	2.91 ± 1.42	4.89 ± 1.89	3.78 ± 1.88	4.16 ± 0.62
(×109/L)	6 month	3.84 ± 1.55	3.34 ± 1.86	3.27 ± 0.97	3.36 ± 1.48
	1 month recovery	1.43 ± 0.91	1.39 ± 0.71	1.50 ± 0.71	0.99 ± 0.42
MONO	3 month	0.40 ± 0.24	0.58 ± 0.20	0.38 ± 0.17	0.53 ± 0.16
(×109/L)	6 month	0.49 ± 0.33	0.32 ± 0.24	0.29 ± 0.14	0.26 ± 0.15*
	1 month recovery	0.12 ± 0.07	0.19 ± 0.15	0.24 ± 0.11	0.15 ± 0.09

*JYG-H, JYG-M, JYG-L* Jueyin granules high-dose, medium dose, low-dose group, respectively, *WBC* total white blood cell count, *RBC* red blood cell, *HGB* hemoglobin, *PLT* blood platelet, *Neut* neutrophil, *Lymph* lymphocyte, *Mono* mononucleosis. The values are expressed as mean ± SEM (n = 10 rats for 3 months; n = 17–19 rats for 6 months; n = 10 rats for 1 month recovery). *Significantly different from control group (*p* < 0.05)

#### Effect of JYG on histopathological alterations of visceral organs

**Fig. 10:**
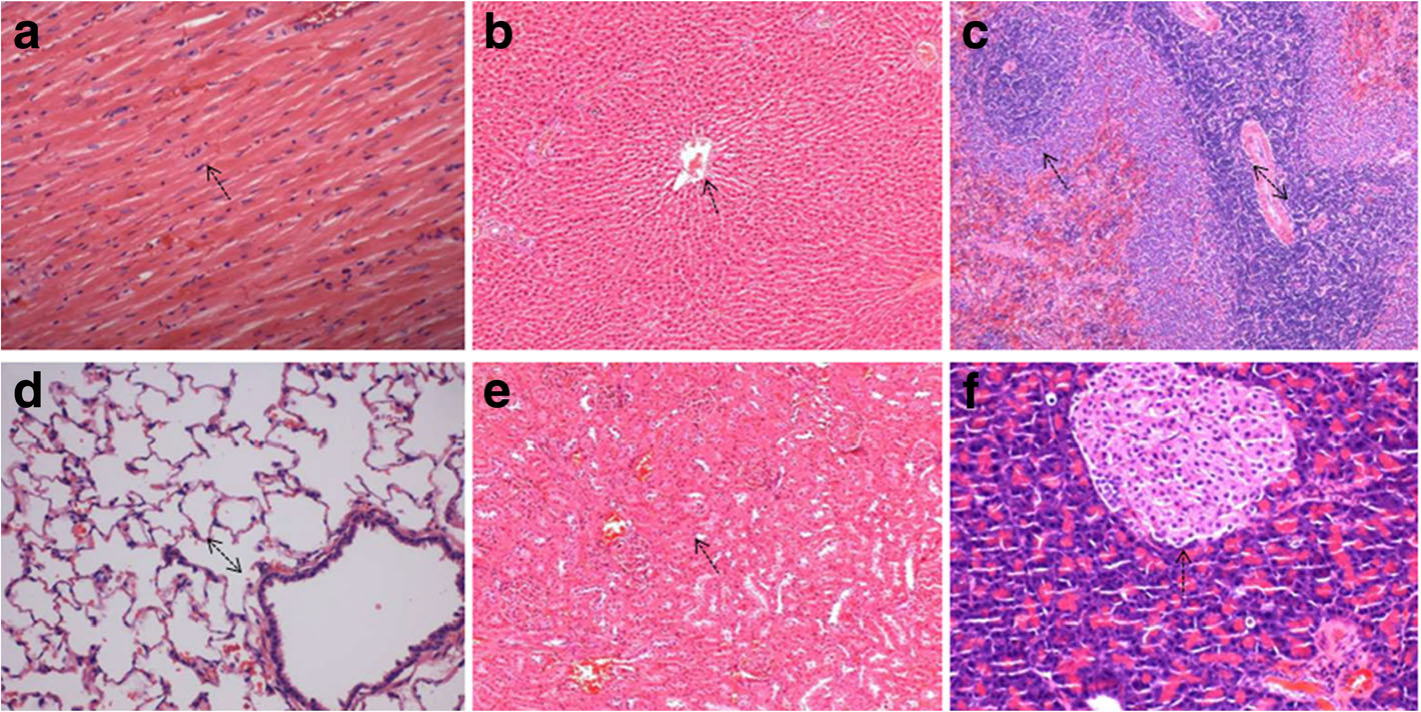
Histopathological analysis of organs stained with H&E. Histopathology showing normal morphology from rats treated with Jueyin granules at the dose of 7.5 g/kg/day. **a** heart, **b** liver, **c** spleen, **d** lung, **e** kidney, **f** pancreas; scale bar = 100 μm

**Fig. 11:**
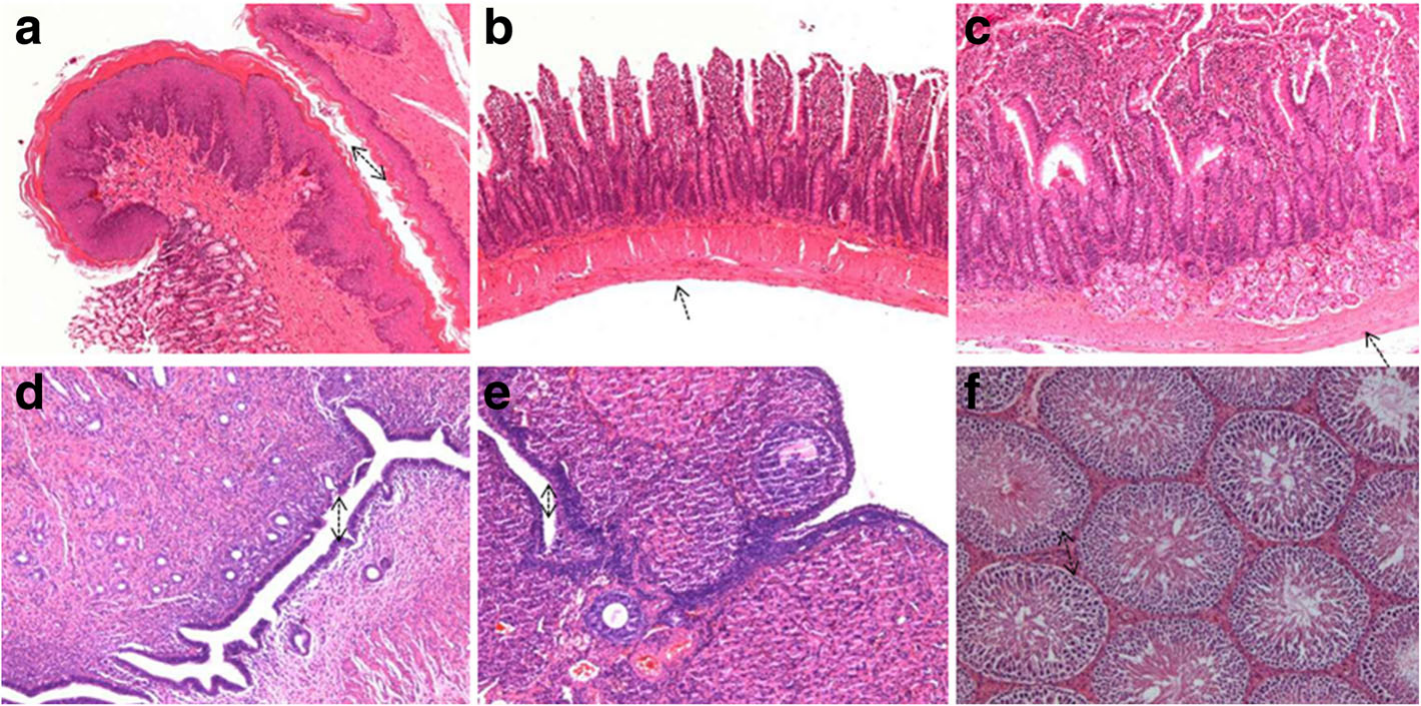
Histopathological analysis of organs stained with H&E. Histopathology showing normal morphology from rats treated with Jueyin granules at the dose of 7.5 g/kg/day. **a** stomach, **b** jejunum, **c** duodenum, **d** uterus, **e** ovaries, **f** orchis; scale bar = 100 μm

**Fig. 12:**
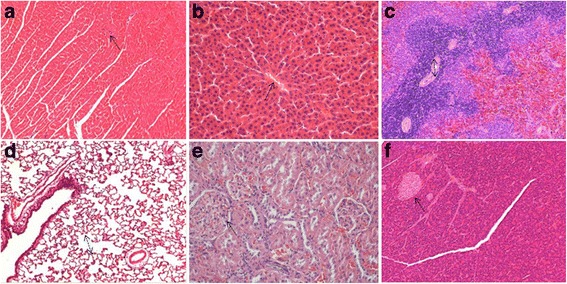
Histopathological analysis of organs stained with H&E. Histopathology showing normal morphology from control group. **a** heart, **b** liver, **c** spleen, **d** lung, **e** kidney, **f** pancreas; scale bar = 100 μm

**Fig. 13:**
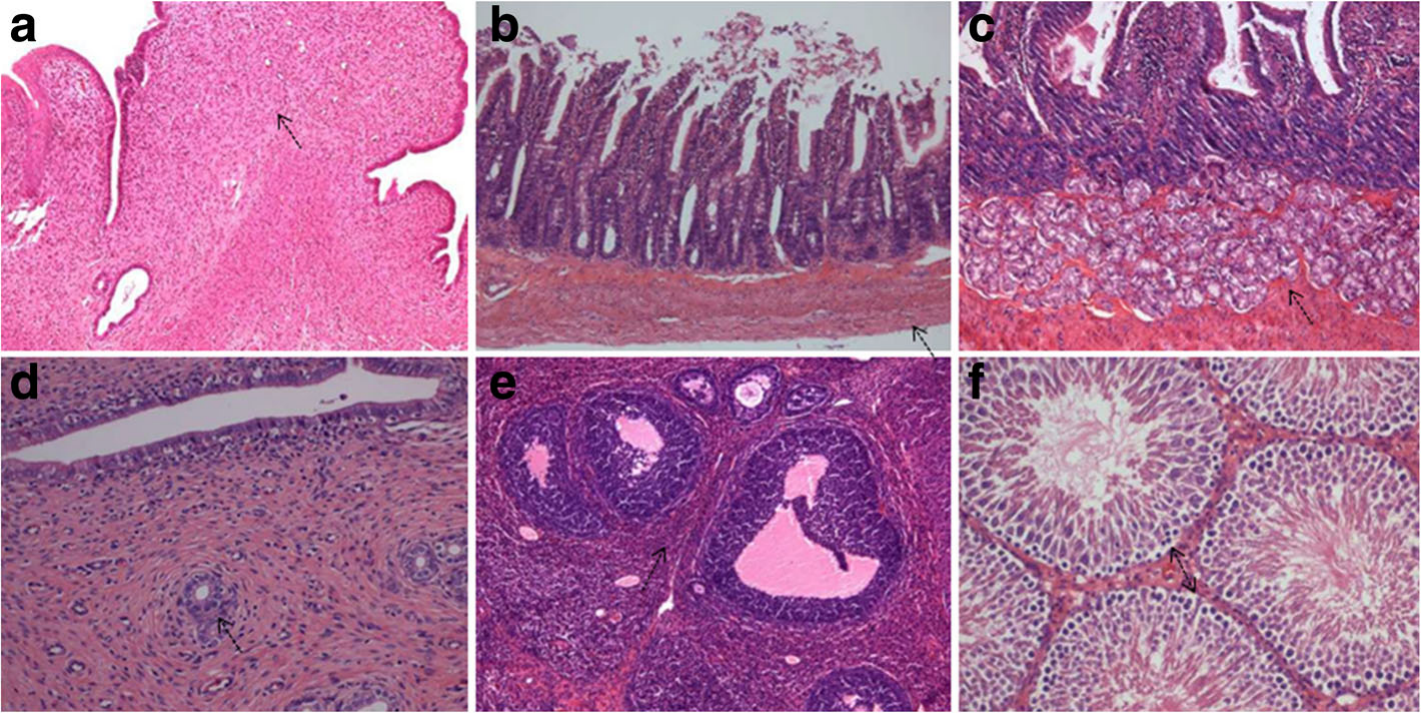
Histopathological analysis of organs stained with H&E. Histopathology showing normal morphology from control group. **a** stomach, **b** jejunum, **c** duodenum, **d** uterus, **e** ovaries, **f** orchis; scale bar = 100 μm

No remarkable gross lesions were detected in any organs of the rats in the JYG-treated groups or those in the control groups. Histopathological examination showed no significant differences in the heart, lung, liver, kidney, spleen, pancreas, stomach, jejunum, duodenum, uterus, ovaries. Orchis between JYG-treated groups and the control group (see Figs. 10, 11, 12 and 13).

## Discussion

Psoriasis, first defined by Ferdinand von Hebra as a distinct entity in 1841, is a common chronic inflammatory skin disorder. It is characterized by abnormal hyperproliferation of epidermal keratinocytes and infiltration of immunocytes along with angiogenesis [14]. The prevalence of psoriasis has been reported to range from 0.91% to 8.5% in adults and from 0 to 2.1% in children [15]. Despite the advances in what is known about the pathogenesis of psoriasis, modern therapies have had limited effects. People are looking for novel drugs to treat psoriasis, but many patients cannot afford the new therapies because of their high cost [16]. Moreover, patients suffering from psoriasis are subjected to considerable physical and psychological disorders,which can aggravate the severity of psoriasis. This situation impairs the quality of life of people with psoriasis [17–19].

Recently, more people with psoriasis have turned to complementary and alternative medicine (CAM) because of its low cost and minimal adverse effects [8]. Traditional Chinese medicine is part of such therapies. Deng et al. [20] reported that herbal formulations could significantly improve the modified psoriasis area severity index score. In addition, these herbs and/or their constituents have anti-inflammatory, anti-angiogenic, anti-proliferative, and tissue repair actions. However, some herbal remedies or herbal formulae can produce a wide range of adverse reactions, even death [21–24]. Therefore, in recent years scientific investigation of CAM has been focused mainly on safety and toxicological evaluations [25].

In the present study, we investigated the acute and chronic toxicity of JYG, which was created in the 1950s by a well-known Chinese surgeon named Han Xia. Although clinical experience and animal studies have demonstrated the effectiveness of JYG in treating psoriasis, some patients suffering from weight loss, gastrointestinal symptom and abnormal liver function were reported in a recent publication [26]. Thus an evaluation on the safety of JYG is necessary.

The acute toxicity test results show that JYG cause no abnormalities or mortality with a maximum dose of 21.5 g/kg, equivalent to 143 times the clinical dose (0.15 g/kg) for a person weighing 60 kg. Therefore JYG could be regarded as a partially nontoxic compound.

In the chronic toxicity test, food consumption and body weight showed a tendency to decrease in the JYG-treated group, especially in male rats. All rats’ weight was recovered at 14 days after the withdrawl of the treatment. Although reduced food intake and weight loss were consistent with clinical observations, there was no significant dose response observed among animals in any of the three groups with different doses. In addition, organ weight and histopathological examinations also remained close to or within the normal range suggesting JYG showed no toxic effects on digestive system in JYG-treated rats. Therefore, we speculate that the reason for the differences is that animals could not adapt to the solution administered. In addition, we recommend that caution still should be taken in determining the dosage of JYG for children.

The serum enzyme levels, organ weight and histopathological examinations remained close to the control values indicating chronic administration of the drug JYG neither impaired the physiology of the liver nor the cellular structures of the liver, which is inconsistent with the clinical observation. However, we speculated the abnormal liver function in the patient may be caused by other reasons such as alcohol consumption rather than JYG administration, since the patient had an alcohol drinking history [26]. No hematological or other biochemistry alterations, or delayed toxic reactions were found in JYG-treated rats. These results were consistent with the recent clinical study showing that patients treated with JYG formulation twice a day for a continuous four-week period had no adverse effects in hematology or hepatorenal functions [26].

## Conclusion

Jueyin granules at the given doses did not produce acute and chronic toxicity in animal models. There were no statistically significant alterations found in behavior, biochemistry, hematological parameters, organ weight, or histopathology. But children, the elderly and those with abnormal digestive function should be used with caution.

## Additional file

Additional file 1: **Figure S1** Histopathological analysis of organs stained with H&E. Histopathology showing normal morphology from the acute toxicity test. (A) heart, (B) liver, (C) spleen, (D) lung, (E) kidney, (F) pancreas; scale bar = 100 μm. **Figure S2** Histopathological analysis of organs stained with H&E. Histopathology showing normal morphology from the acute toxicity test. (A) stomach, (B) jejunum, (C) duodenum, (D) uterus, (E) ovaries, (F) orchis; scale bar = 100 μm. **Figure S3** Histopathological analysis of organs stained with H&E. Histopathology showing normal morphology from eight dead rats. (A) heart, (B) liver, (C) spleen, (D) lung, (E) kidney, (F) pancreas; scale bar = 100 μm. **Figure S4** Histopathological analysis of organs stained with H&E. Histopathology showing normal morphology from eight dead rats. (A) stomach, (B) jejunum, (C) duodenum, (D) uterus, (E) ovaries, (F) orchis; scale bar = 100 μm (PPTX 1332 kb)

## Abbreviations

ALT: alanine aminotransferase
AST: aspartate aminotransferase
BUN: blood urea nitrogen
CAM: complementary and alternative medicine
Cl^−^: Chloride ion
CRE: creatinine
HGB: hemoglobin
JYG: Jueyin granules
JYG-H: Jueyin granules high-dose group
JYG-L: Jueyin granules low-dose group
JYG-M: Jueyin granules medium-dose group
K^+^: Potassium ion
Lymph: lymphocyte
Mono: mononucleosis
Na^+^: Sodium ion
Neut: neutrophil
PLT: blood platelet
RBC: red blood cell count
ROW: relative organs weight
WBC: total white blood cell count
