# Supplementary material for: Acute and chronic toxicity of a polyherbal preparation – Jueyin granules
Source: BMC Complement Altern Med. 2018 May 8;18:148. doi: 10.1186/s12906-018-2211-z (PMC5941322; doi:10.1186/s12906-018-2211-z)
Supplement: Supplementary file 1 — Figure S1 Histopathological analysis of organs stained with H&E. Histopathology showing normal morphology from the acute toxicity test. (A) heart, (B) liver, (C) spleen, (D) lung, (E) kidney, (F) pancreas; scale bar = 100 μm. Figure S2 Histopathological analysis of organs stained with H&E. Histopathology showing normal morphology from the acute toxicity test. (A) stomach, (B) jejunum, (C) duodenum, (D) uterus, (E) ovaries, (F) orchis; scale bar = 100 μm. Figure S3 Histopathological analysis of organs stained with H&E. Histopathology showing normal morphology from eight dead rats. (A) heart, (B) liver, (C) spleen, (D) lung, (E) kidney, (F) pancreas; scale bar = 100 μm. Figure S4 Histopathological analysis of organs stained with H&E. Histopathology showing normal morphology from eight dead rats. (A) stomach, (B) jejunum, (C) duodenum, (D) uterus, (E) ovaries, (F) orchis; scale bar = 100 μm (PPTX 1332 kb) [file 12906_2018_2211_MOESM1_ESM.pptx]

## Slide 1
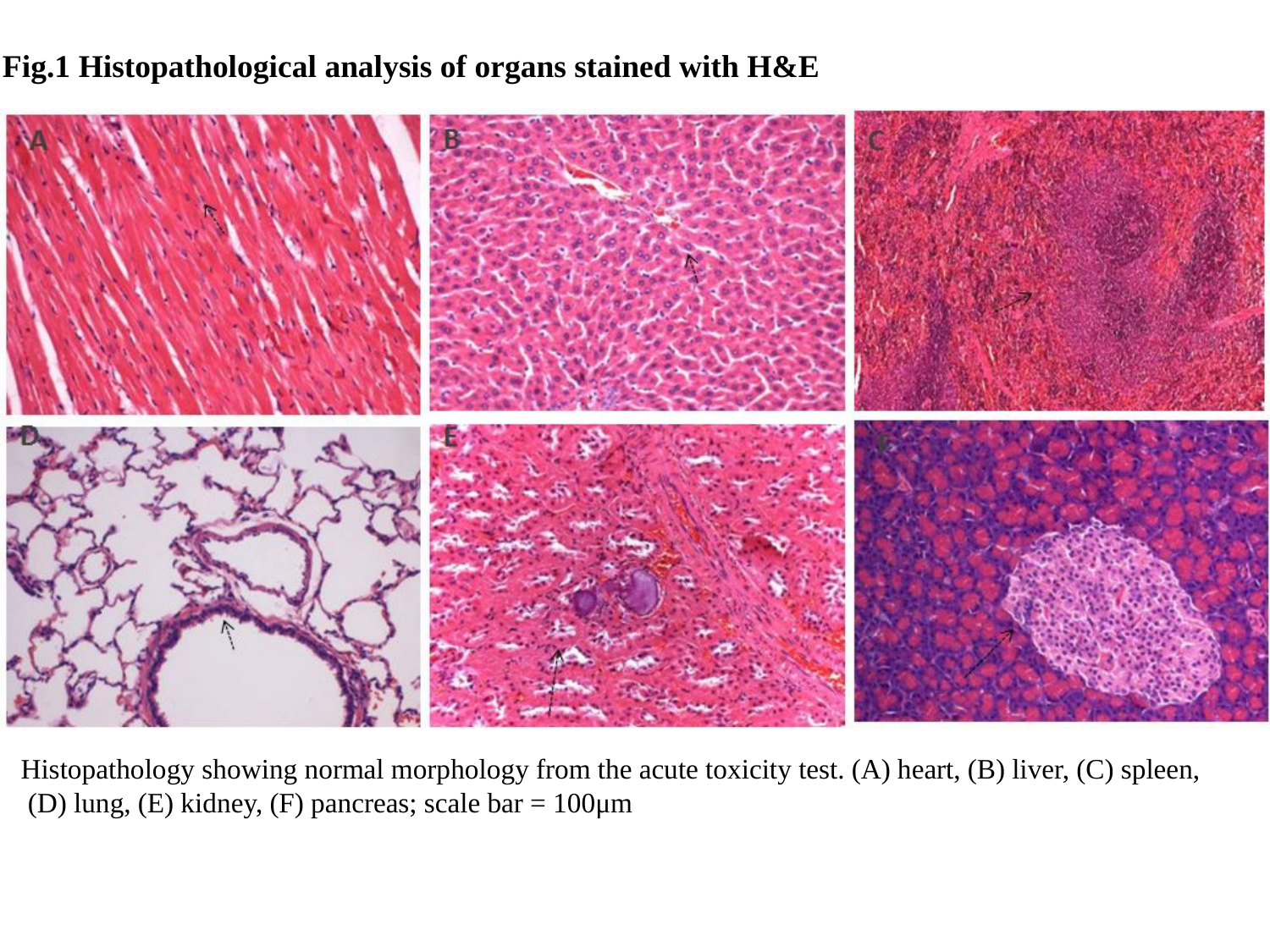

Fig.1 Histopathological analysis of organs stained with H&E
Histopathology showing normal morphology from the acute toxicity test. (A) heart, (B) liver, (C) spleen,
 (D) lung, (E) kidney, (F) pancreas; scale bar = 100μm

## Slide 2
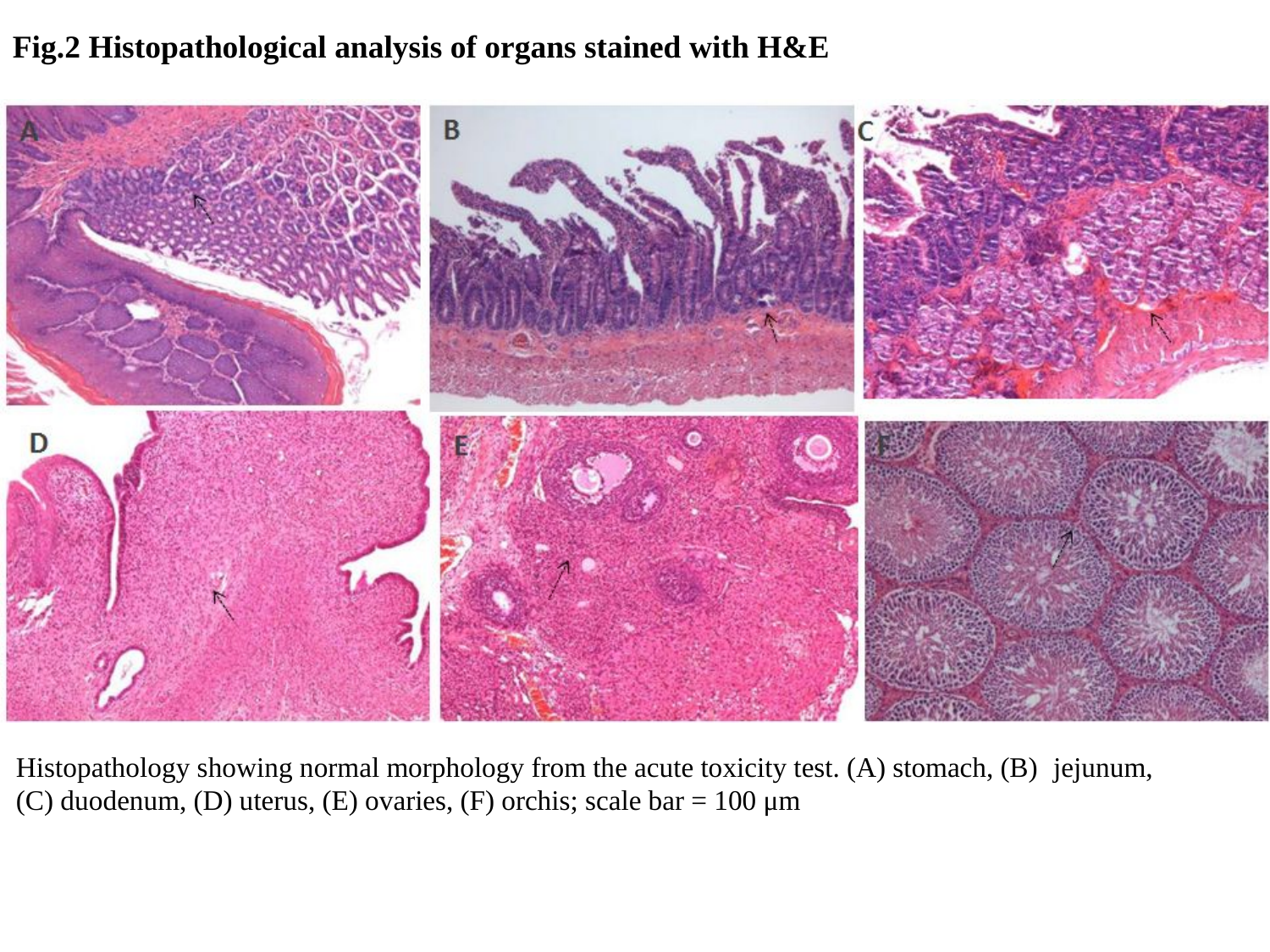

Fig.2 Histopathological analysis of organs stained with H&E
Histopathology showing normal morphology from the acute toxicity test. (A) stomach, (B)  jejunum,
(C) duodenum, (D) uterus, (E) ovaries, (F) orchis; scale bar = 100 μm

## Slide 3
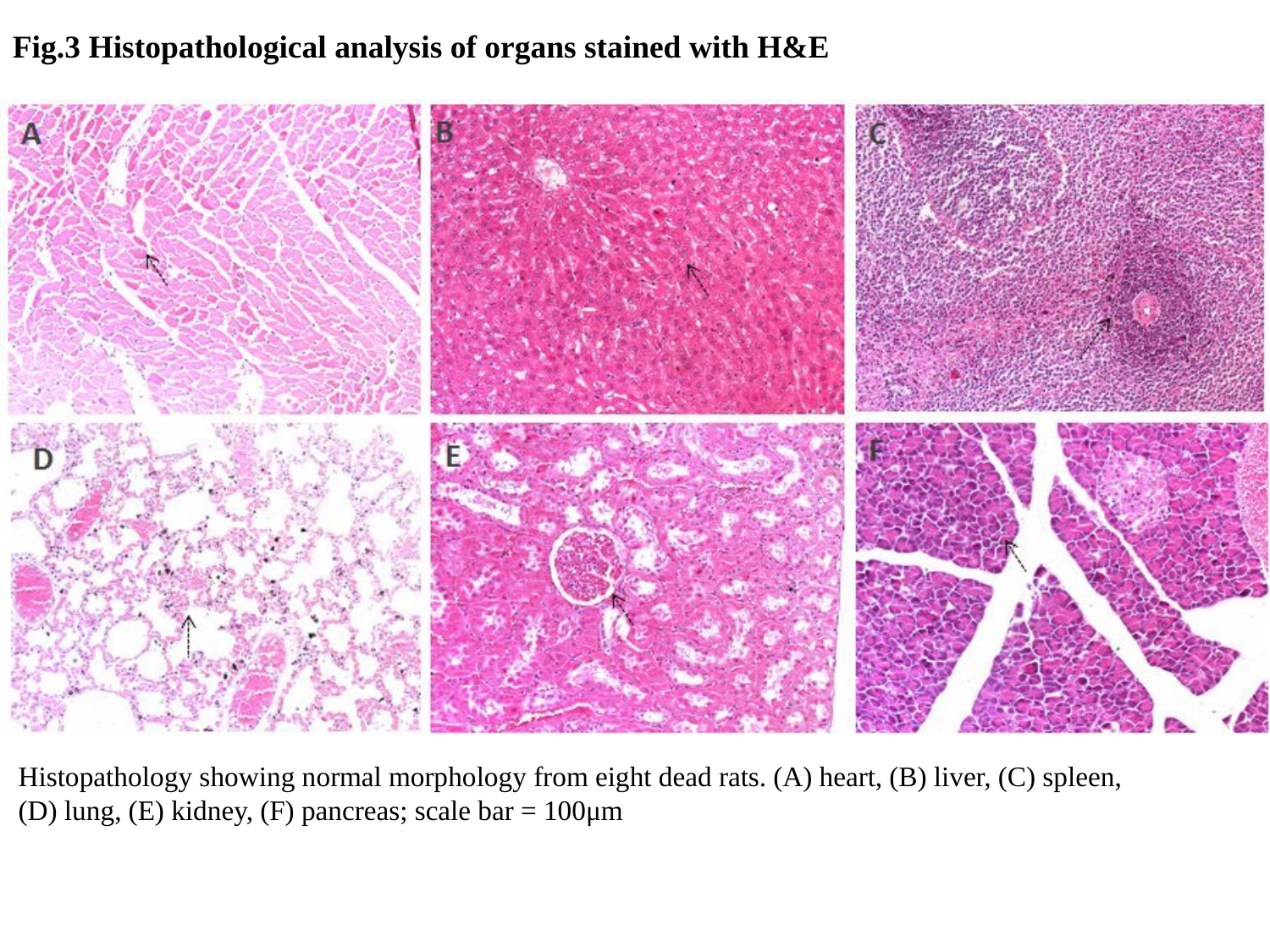

Fig.3 Histopathological analysis of organs stained with H&E
Histopathology showing normal morphology from eight dead rats. (A) heart, (B) liver, (C) spleen,
(D) lung, (E) kidney, (F) pancreas; scale bar = 100μm

## Slide 4
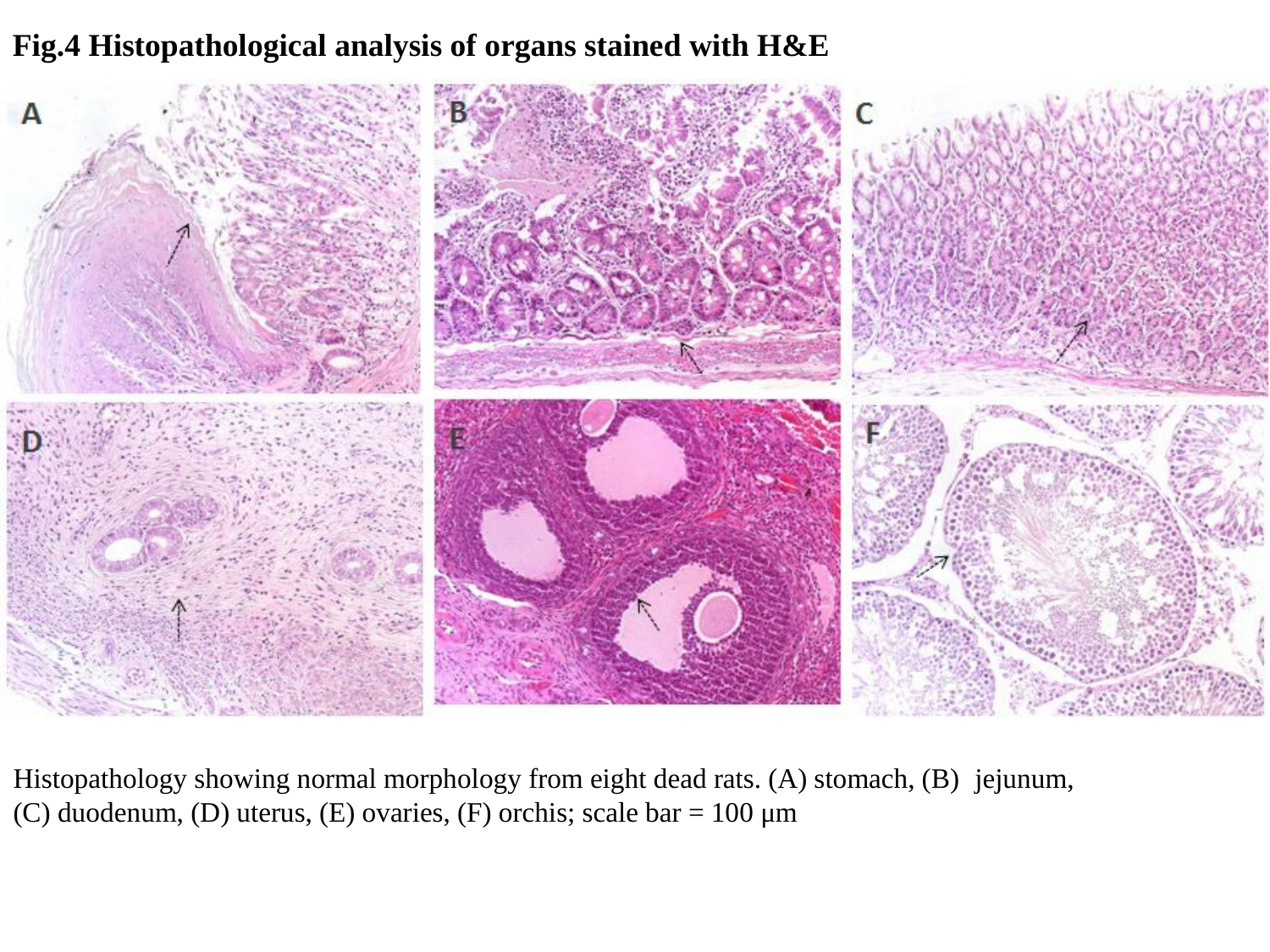

Fig.4 Histopathological analysis of organs stained with H&E
Histopathology showing normal morphology from eight dead rats. (A) stomach, (B)  jejunum,
(C) duodenum, (D) uterus, (E) ovaries, (F) orchis; scale bar = 100 μm
